# Impact of e-Health Interventions on Mental Health and Quality of Life in Breast Cancer Patients: A Systematic Review and Meta-Analysis of Randomized Controlled Trials

**DOI:** 10.3390/cancers17111780

**Published:** 2025-05-26

**Authors:** Alexandros Mitsis, Panagiotis Filis, Georgia Karanasiou, Eleni I. Georga, Davide Mauri, Katerina K. Naka, Anastasia Constantinidou, Kalliopi Keramida, Dorothea Tsekoura, Ketti Mazzocco, Alexia Alexandraki, Effrosyni Kampouroglou, Yorgos Goletsis, Andri Papakonstantinou, Athos Antoniades, Cameron Brown, Vasileios Bouratzis, Erika Matos, Kostas Marias, Manolis Tsiknakis, Dimitrios I. Fotiadis

**Affiliations:** 1 Unit of Medical Technology and Intelligent Information Systems, Department of Material Science and Engineering, University of Ioannina, 45110 Ioannina, Greece; alexmitsis07@gmail.com (A.M.); g.karanasiou@gmail.com (G.K.); egewrga@gmail.com (E.I.G.); goletsis@uoi.gr (Y.G.); 2 Department of Hygiene and Epidemiology, University of Ioannina School of Medicine, 45110 Ioannina, Greece; png.filis@gmail.com; 3 Department of Medical Oncology, University of Ioannina, 45110 Ioannina, Greece; dvd.mauri@gmail.com; 4 Department of Biomedical Research, Institute of Molecular Biology and Biotechnology, FORTH, 45110 Ioannina, Greece; 5 Second Department of Cardiology, University Hospital of Ioannina, Stavros Niarchos Avenue, 45500 Ioannina, Greece; anaka@uoi.gr (K.K.N.); v.bouratzis@gmail.com (V.B.); 6 Bank of Cyprus Oncology Centre, Nicosia 2029, Cyprus; anastasia.constantinidou@bococ.org.cy; 7 Cyprus Cancer Research Institute, Nicosia 2109, Cyprus; 8 Medical School, University of Cyprus, Nicosia 2029, Cyprus; 9 General Anti-Cancer Oncological Hospital, Agios Savvas, 11522 Athens, Greece; keramidakalliopi@hotmail.com; 10 Department of Cardiology, University Hospital Attikon, National and Kapodistrian University of Athens, 12462 Athens, Greece; 11 2nd Department of Surgery, Aretaieio University Hospital, National and Kapodistrian University of Athens, 76 Vas. Sofias Av., 11528 Athens, Greece; dtsekoura@hotmail.com (D.T.); efrosini.kampouroglou@gmail.com (E.K.); 12 European Institute of Oncology IRCCS, 20141 Milan, Italy; ketti.mazzocco@ieo.it; 13 Department of Oncology and Hemato-Oncology, University of Milan, 20122 Milan, Italy; 14 A.G. Leventis Clinical Trials Unit, Bank of Cyprus Oncology Centre, 32 Acropoleos Avenue, Nicosia 2006, Cyprus; alexia.alexandraki@bococ.org.cy; 15 Laboratory of Business Economics and Decisions (LABED@UoI), Department of Economics, University of Ioannina, 45110 Ioannina, Greece; 16 Department of Oncology-Pathology, Karolinska Institutet and University Hospital, 171 64 Stockholm, Sweden; andri.papakonstantinou@ki.se; 17 Stremble Ventures Ltd., 59 Christaki Kranou, Limassol 4042, Cyprus; athos.antoniades@stremble.com (A.A.); cameron.brown@stremble.com (C.B.); 18 Department of Medical Oncology, Institute of Oncology Ljubljana, 1000 Ljubljana, Slovenia; ematos@onko-i.si; 19 Department of Electrical and Computer Engineering, Hellenic Mediterranean University, 71410 Heraklion, Greece; kmarias@ics.forth.gr (K.M.); tsiknaki@hmu.gr (M.T.); 20 Computational Biomedicine Laboratory, Institute of Computer Science, FORTH, 70013 Heraklion, Greece

**Keywords:** e-health, breast cancer, anxiety, depression, quality of life

## Abstract

**Background/Objectives:** The prevalence of breast cancer (BC) is significant globally. The malignancy itself and the related treatments have a considerable impact on patients’ overall well-being. The adoption of e-health solutions for patients is increasing rapidly worldwide, since these innovative tools hold significant potential to positively impact the mental health and quality of life (QoL) of BC patients. However, their overall impact is still being explored, and further understanding and analysis are required. This review paper aims to present, quantify, and summarize the cumulative available randomized evidence on the state of the art of supportive interventions delivered via e-health applications for patients’ mental health and QoL before, during, and after BC treatment. **Methods**: A systematic review was conducted following the PRISMA guidelines in the Scopus and PubMed databases on 7 November 2024 to identify studies that utilized internet-based interventions in BC patients. The inclusion criteria were as follows: adult men and women (aged > 18 years) diagnosed with breast cancer (BC) who received patient-directed e-health interventions, compared to standard care or control interventions. The studies had to focus on outcomes such as quality of life (QoL), anxiety, depression, and distress, and be limited to randomized controlled trials (RCTs). The PRISMA-P guidelines were followed. Risk of bias was assessed using the Cochrane risk-of-bias (RoB) tool for randomized controlled trials. **Results**: A total of 27 randomized studies, involving 2898 patients, were included in this systematic review. The e-health interventions significantly affected patients’ anxiety (SMD = −0.80; 95% CI: −1.33 to −0.27; *p* < 0.01; and I^2^ = 94%), depression (SMD = −0.74; 95% CI: −1.40 to −0.09; *p* = 0.026; and I^2^ = 95%) and QoL (SMD = 0.65; 95% CI: 0.27 to 1.04; *p* < 0.01; and I^2^ = 90%) but had no significant effect on distress (SMD = −0.78; 95% CI: −1.93 to 0.37; *p* = 0.184; and I^2^ = 95%). **Conclusions**: This study showed that e-health interventions can improve QoL, reduce anxiety, and decrease depression in adult BC patients. However, no noticeable impact on reducing distress levels was observed. Additionally, given the diversity of interventions, these results should be interpreted with caution. To determine the optimum duration, validate different intervention approaches, and address methodological gaps in previous studies, more extensive clinical studies are needed.

## Simple Summary

Breast cancer is widespread globally and significantly affects patients’ well being. e-Health solutions are rapidly increasing, offering support for patients’ mental health and quality of life. This systematic review includes 27 randomized studies with a total of 2898 patients, which evaluated the effects of e-Health interventions on mental health and quality of life in breast cancer patients. The results show a significant reduction in anxiety and depression and an improvement in quality of life, but no significant effect on reducing distress.

## 1. Introduction

Breast cancer (BC) is the most common type of cancer, commonly diagnosed in women, although it can occur in men [1]. It poses a significant health burden globally, with over 2 million new cases diagnosed in 2022 [2]. It is also one of the leading causes of death in women worldwide, despite its downward trend, particularly in developed countries. This highlights the importance of advancing BC management, with a focus on enhancing early detection methods and developing more effective treatment options [3].

Currently, therapeutic options for BC encompass surgical interventions, chemotherapy, endocrine therapy, radiotherapy, targeted therapy, and immunotherapy [4]. The evolution of novel treatments and therapies has notably improved the survival of BC patients. Approximately 70% of patients experience an increase in life expectancy of more than five years, while 40% experience an increase of more than ten years. Moreover, for 15% of patients, life expectancy extends by over twenty years [5]. However, it is well recognized that patients’ mental health and QoL are typically impacted by BC treatment and its sequelae, and some of them can be lifelong [6]. Thus, the transition from the “cancer struggle” to “regular life” for survivors mandates that they first deal with the adverse effects of cancer therapy [7]. Therefore, as survival rates have improved considerably [5], the mitigation of cancer therapy-related adverse effects is crucial to improving QoL. The management of QoL, in its physical and psychological components, will result in improved treatment efficacy and prognosis [8].

E-health refers to the use and application of digital technologies (e.g., internet, mobile devices, wearables, software tools, etc.) that support healthcare delivery for improved disease monitoring, management, and QoL [9]. The rapid improvements and increasing accessibility of these technologies have driven the widespread adoption of e-health interventions in cancer care, enabling, among others, patient engagement and communication with healthcare experts throughout the healthcare delivery continuum [10]. Currently, there are several studies showing that e-health interventions may have a positive effect on cancer patients’ physical, psychological, and social functioning, as well as their self-efficacy, QoL, mental well-being, depression, and anxiety [5,11,12,13]. Nonetheless, the overall impact of e-health interventions on patients’ mental health and QoL is not yet clear, largely due to the variability in study designs, intervention types, and outcome measures used in existing research, and this variability presents a significant challenge in conducting comprehensive reviews to accurately estimate their overall effectiveness.

To address this challenge, we performed a systematic review with the aim of quantifying and summarizing the available randomized evidence on the use of supportive interventions delivered via e-health on patients’ mental health and QoL.

## 2. Materials and Methods

### 2.1. Search Strategy

The PubMed and Scopus databases were systematically scrutinized from inception up to 7 November 2024 for eligible studies. This study was registered in the International Prospective Register of Systematic Reviews.

To identify relevant studies for this review, a Boolean string consisting of several relevant keywords was generated. The following string was applied: “((quality of life) AND (mental health AND ((mental OR emotional OR psychological OR social) AND well-being) OR mental disorder OR depression OR anxiety) AND (breast cancer) AND (e-health OR electronic health OR information and communication technolog* OR ICT OR m-health OR mobile health OR digital health OR mobile OR internet OR web OR online OR digital OR remote OR smartphone OR application OR app OR e-coach))”. This string was developed so that the search of each database only identified studies relevant to the topic, thereby ensuring consistency and comprehensiveness in the search process. For a study to be considered for inclusion in our analysis, it had to meet the predefined inclusion criteria listed in Table 1. In addition, the study had to be published in English, and the full text had to be available. The inclusion criteria were determined using the PICOS framework [14].

### 2.2. Data Extraction

The papers retrieved were subsequently handled with an automated tool (Zotero 6.0.36, Corporation for Digital Scholarship, Vienna, VA, USA), which was used to remove duplicate entries. The remaining articles were then independently screened for title and abstract by each of the two reviewers who participated in the study selection process. Potentially eligible articles were then assessed in full text by the same reviewers independently once again. Any disagreements were resolved through discussion or, if necessary, with the involvement of a third reviewer.

The data from the remaining studies were extracted by two independent reviewers according to the PRISMA guidelines.

A predefined data extraction sheet was used to collect information from each study. The focus was on evaluating changes in anxiety, depression, quality of life, and distress before and after the intervention involving e-health applications. Studies that did not report outcomes for any of these categories were excluded from the current analysis. Data extraction was performed independently by two authors, and the accuracy of the extracted data was verified by a third author. For each study, we extracted information regarding the authors, publication year, ID, sample size, cancer and therapy information, intervention type, duration of the intervention, study design, outcomes of interest, and a summary of the results.

### 2.3. Risk of Bias and Quality Assessment

Two independent reviewers assessed the quality of the included studies using the Cochrane risk-of-bias tool, a commonly used method for assessing the risk of bias in various study designs, including randomized controlled trials (RCTs) [15]. The quality of each study was evaluated in the following domains: adequate sequence generation, allocation concealment, adequate blinding of patients and personnel, adequate blinding of outcome assessors, incomplete outcome data assessment, and selective reporting bias [16].

### 2.4. Statistical Analysis

We performed a meta-analysis using RStudio software (version 4.3.1; R Core Team, 2023) with the ‘meta’ and ‘metafor’ packages. Standardized mean differences (SMDs) and 95% confidence intervals were used for the outcomes, measured as the mean and standard deviation. A random-effects model was employed to account for potential differences in samples and interventions across the included studies. A *p*-value of <0.05 was considered statistically significant. Statistical heterogeneity was assessed using the I^2^ statistic [17]. Values of 0–25%, 25–50%, and 50–100% were considered to indicate low, moderate, and substantial heterogeneity, respectively [18]. Moreover, subgroup analyses were performed based on the mode of e-health intervention (Web, mobile applications, other), as well as the duration of the intervention (less than 12 weeks and 12 weeks or more). Small study effects, indicative of publication bias, were assessed by visual inspection of funnel plots and Egger’s test [19]. This assessment was performed only for analyses that included 10 or more studies, and a *p*-value < 0.1 was considered indicative of small study effects.

## 3. Results

**Figure 1:**
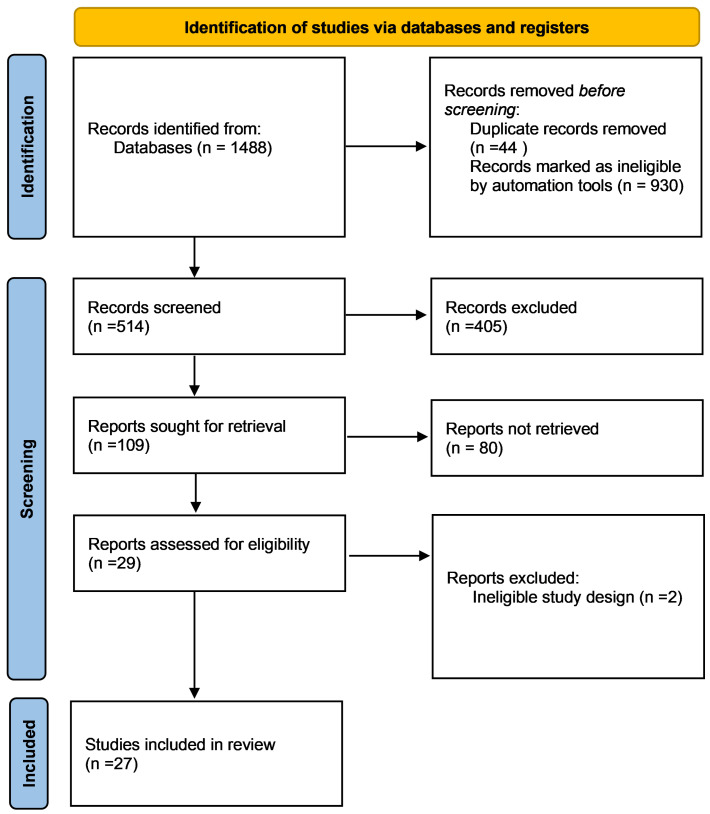
PRISMA flow diagram.

A total of 1488 records were identified following the search of Scopus and PubMed. After title and abstract screening, 109 publications were identified as potentially eligible (Figure 1). Following detailed screening, our systematic review retained a total of 27 studies focusing on e-health interventions targeting mental health and QoL in BC patients.

### 3.1. Characteristics of Included Studies

All the studies included in the final analysis focused on adult patients and cancer survivors aged 18 years or older. All the studies included were RCTs specifically targeting patients diagnosed with BC. The sample size ranged from 35 to 363 patients. Five studies had a sample size of less than 50 patients, twelve between 50 and 100 patients, while the remaining studies had a sample size of more than 100 patients. In total, (i) patients in 10 studies had a mean age between 50 and 60 years, patients in 1 study had a mean age of over 60 years, and patients in the remaining studies had a mean age of 50 years or younger; (ii) the publications were from 2018 onward: nine studies were published in 2024, two studies in 2023, three studies in 2022, two studies in 2021, four studies in 2020, three studies in 2019, and four studies in 2018; and (iii) the majority (13/27) were conducted in Asia, followed by seven, four, and three in Europe, America, and Australia, respectively.

The included studies had different intervention durations, ranging from 3 to 24 weeks, and different e-health tools were used. Notably, the majority of studies (16/27) used mHealth apps [20,21,22,23,24,25,26,27,28,29,30,31,32,33,34,35,36,37], while the remaining studies used web applications [38,39,40,41,42,43,44,45,46].

The main characteristics and findings of the studies included are summarized in Table 2, Table 3 and Table 4.

### 3.2. Risk of Bias Within Studies

We used the Cochrane risk-of-bias tool to assess the quality of the included studies. Of the 27 included studies, only three RCTs met all the requirements to be considered as having a low risk of bias. Of the 27 included studies, 24 (89%) had an appropriate sequence generation process, while only 13 used allocation concealment. Most of the studies (81.5%) did not blind their patients or professionals, or it was unclear whether blinding was used, resulting in a high or unclear risk of bias. Thirteen studies (48%) implemented blinding of outcome assessors. Twenty studies (74%) were judged as posing a low risk of incomplete outcome data.

**Figure 2:**
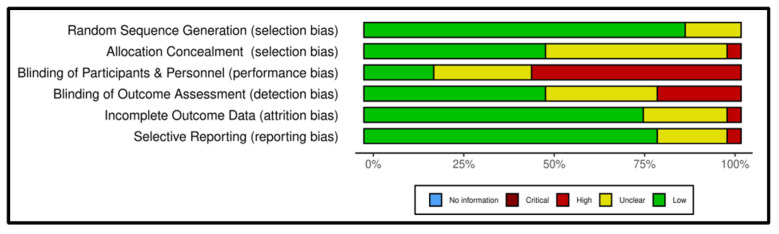
Risk-of-bias graph.

**Figure 3:**
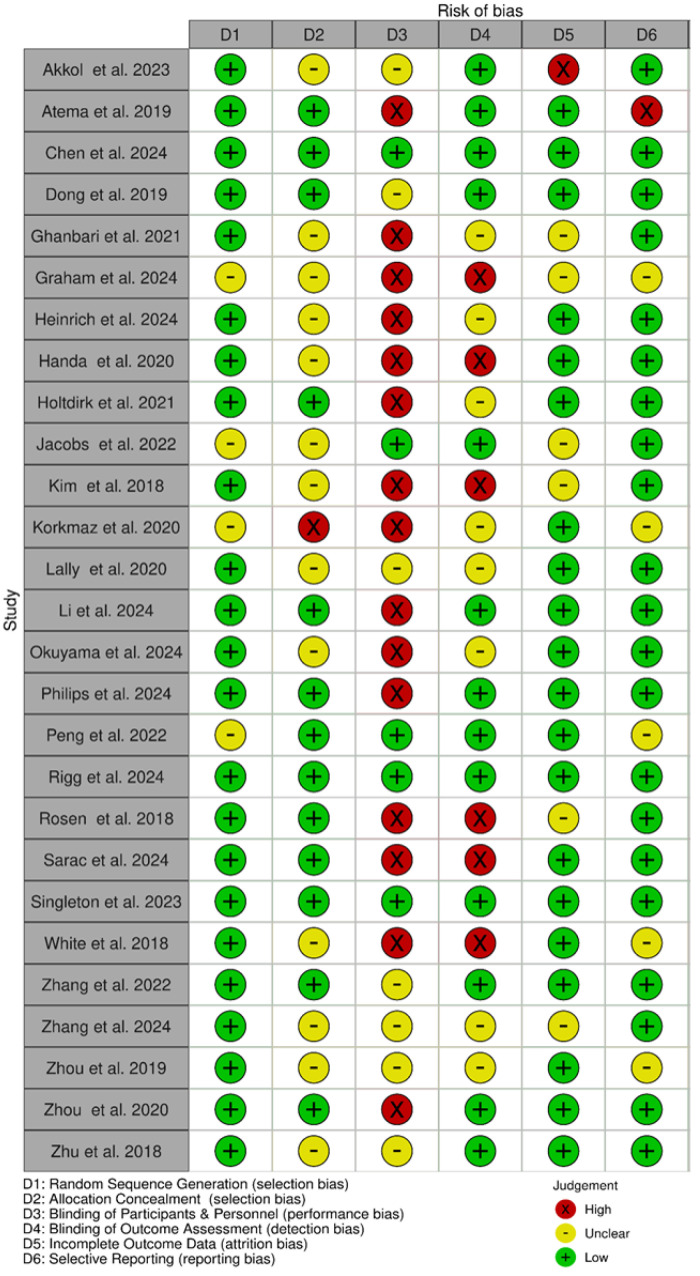
Risk-of-bias summary [20,21,22,23,24,25,26,27,28,29,30,31,32,33,34,35,36,37,38,39,40,41,42,43,44,45,46].

Altogether, 21 (78%) studies were considered as having a low risk of reporting bias. Figure 2 and Figure 3 show the results of the quality assessment of the included studies using the Cochrane Collaboration’s tool.

### 3.3. Intervention Outcomes

#### 3.3.1. Anxiety

**Figure 4:**
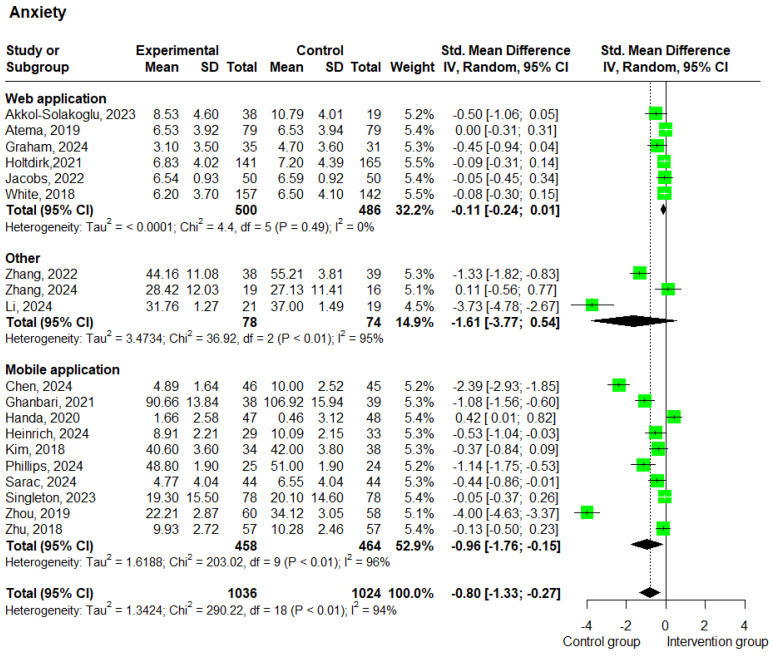
Forest plots—effects of e-health interventions on anxiety, presented by method of intervention (web application: *p* = 0.083; mobile application: *p* = 0.019; other: *p* = 0.142) [20,22,23,24,25,27,30,31,33,34,35,36,37,38,39,40,41,45,46]. SD, standard deviation; SMD, standardized mean difference. The “Other” category includes two studies that used virtual reality-based interventions and one study that used a wearable-based intervention.

Nineteen studies [20,22,23,24,25,27,30,31,33,34,35,36,37,38,39,40,41,45,46] provided data on anxiety for a total of 2060 patients. Heterogeneity testing showed I^2^ = 94%, indicating high heterogeneity among the studies. The results of the analysis showed that the anxiety levels of the control group were higher than those of the intervention group (SMD = −0.80; 95% CI: −1.33 to −0.27; *p* < 0.01; and I^2^ = 94%) (Figure 4).

The subgroup analysis based on the delivery method showed that studies using web-based interventions (*n* = 6) [38,39,40,41,45,46] yielded an SMD of −0.11 (95% CI: −0.24 to 0.01; *p* = 0.083; and I^2^ = 0%), while studies using mobile-based interventions (*n* = 10) [20,22,23,24,27,30,31,33,34,35] showed a statistically significant SMD of −0.96 (95% CI: −1.76 to −0.15; *p* = 0.019; and I^2^ = 96%) (Figure 4). The subgroup analysis based on the intervention duration showed a statistically significant decrease in anxiety in the intervention group for both categories, with studies with a duration of less than 12 weeks (*n* = 5) [20,22,30,35,46] showing an SMD of −0.95 (95% CI: −1.69 to −0.21; *p* = 0.011; and I^2^ = 90%), and studies lasting 12 weeks or more (*n* = 14) [23,24,25,27,31,33,34,36,37,38,39,40,41,45] showing an SMD of −0.75 (95% CI: −1.45 to −0.06; *p* = 0.033; and I^2^ = 94%) (Supplementary Figure S1). It is important to note that low scores on the anxiety scales correspond to a lower level of anxiety.

#### 3.3.2. Depression

**Figure 5:**
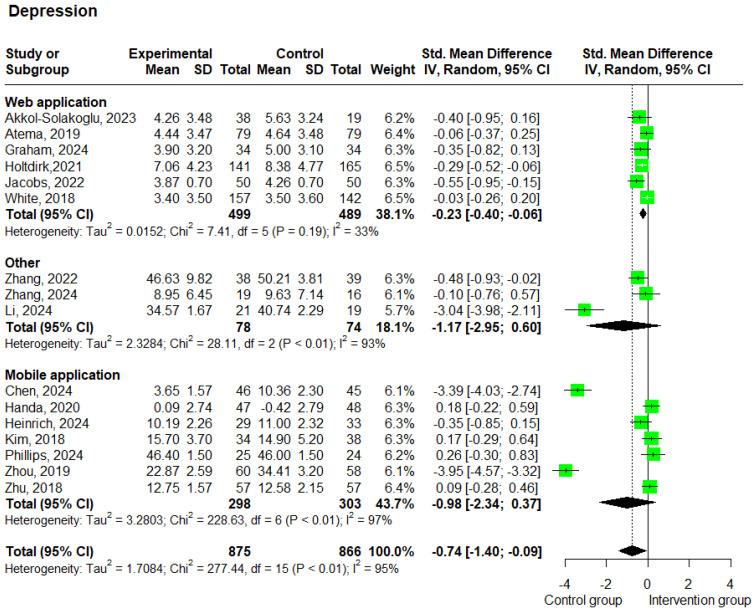
Forest plots—effects of e-health interventions on depression, presented by method of intervention (web application: *p* = 0.008; mobile application: *p* = 0.155; other: *p* = 0.195) [20,23,24,25,27,33,34,35,36,37,38,39,40,41,45,46]. SD, standard deviation; SMD, standardized mean difference. The “Other” category includes two studies that used virtual reality-based interventions and one study that used a wearable-based intervention.

Sixteen studies [20,23,24,25,27,33,34,35,36,37,38,39,40,41,45,46] involving 1741 patients reported depression outcomes. The meta-analysis results showed that patients receiving e-health interventions reported lower levels of depression compared to the control group and that this difference was statistically significant (SMD −0.74; 95% CI −1.40 to −0.09; *p* = 0.026; and I^2^ = 95%). The heterogeneity test showed a substantial I^2^ = 95%, indicating high heterogeneity among the studies (Figure 5).

The subgroup analysis based on the delivery method showed that studies using web-based interventions (*n* = 6) [38,39,40,41,45,46] yielded a statistically significant SMD of −0.23 (95% CI: −0.40 to −0.06; *p* = 0.008; and I^2^ = 33%), while studies using mobile-based interventions (*n* = 7) [20,23,24,27,33,34,35] reported an SMD of −0.98 (95% CI: −2.34 to 0.37; *p* = 0.155; and I^2^ = 97%) (Figure 5). Furthermore, the subgroup analysis based on the intervention duration revealed that studies with a duration of less than 12 weeks (*n* = 3) [20,35,46] showed an SMD of −1.19 (95% CI: −3.35 to 0.96; *p* = 0.278; and I^2^ = 97%), while studies lasting 12 weeks or more (*n* = 13) [23,24,25,27,33,34,36,37,38,39,40,41,45] showed an SMD of -0.64 (95% CI: −1.31 to 0.04; *p* = 0.065; and I^2^ = 94%) (Supplementary Figure S2). Additionally, it is important to note that low scores on the depression scales correspond to a lower level of depression.

#### 3.3.3. QoL

**Figure 6:**
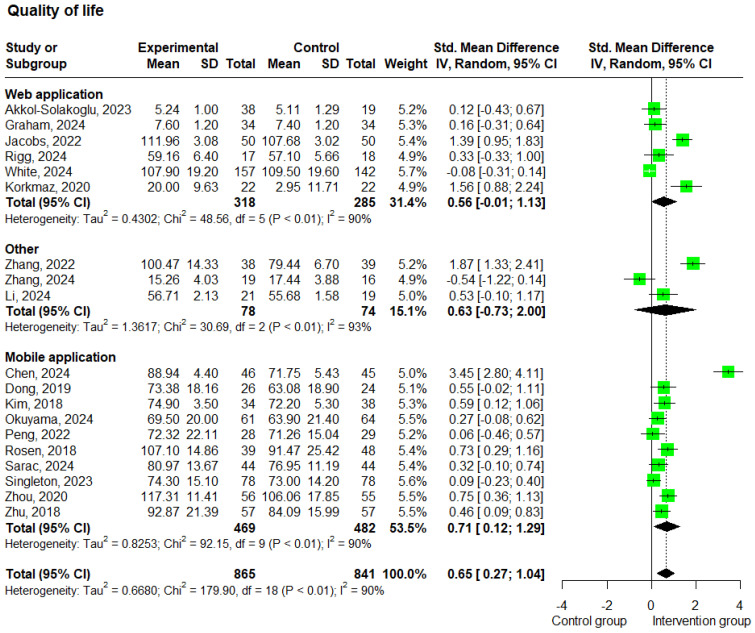
Forest plots—effects of e-health interventions on QoL, presented by method of intervention (web application: *p* = 0.052; mobile application: *p* = 0.017; other: *p* = 0.364) [20,21,25,26,28,29,30,31,32,33,35,36,37,39,41,42,44,45,46]. SD, standard deviation; SMD, standardized mean difference. The “Other” category includes two studies that used virtual reality-based interventions and one study that used a wearable-based intervention.

A total of 19 studies [20,21,25,26,28,29,30,31,32,33,35,36,37,39,41,42,44,45,46] reported data on QoL. These studies included 1,706 patients and showed substantial heterogeneity (I^2^ = 90%). Compared to the control group, the analysis of patients receiving e-health interventions showed better QoL, and this difference was statistically significant (SMD: 0.65; 95% CI: 0.27 to 1.04; *p* < 0.01; and I^2^ = 90%) (Figure 6).

The subgroup analysis based on the delivery method showed that studies using web-based interventions (*n* = 6) [39,41,42,44,45,46] yielded an SMD of 0.56 (95% CI: −0.01 to 1.13; *p* = 0.052; and I^2^ = 90%), while studies using mobile-based interventions (*n* = 10) [20,21,26,28,29,30,31,32,33,35] reported a statistically significant SMD of 0.71 (95% CI: 0.12 to 1.29; *p* = 0.017; and I^2^ = 90%) (Figure 6). Furthermore, the subgroup analysis based on the intervention duration in QoL studies revealed that studies with a duration of less than 12 weeks (*n* = 7) [20,29,30,35,42,44,46] showed a statistically significant SMD of 1.00 (95% CI: 0.15 to 1.86; *p* = 0.022; and I^2^ = 93%), while studies lasting 12 weeks or more (*n* = 12) [21,25,26,28,31,32,33,36,37,39,41,45] showed a statistically significant SMD of 0.46 (95% CI: 0.10 to 0.81; *p* = 0.012; and I^2^ = 87%) (Supplementary Figure S3). Moreover, higher scores on the QoL scales indicate a higher level of QoL for the patients.

#### 3.3.4. Distress

**Figure 7:**
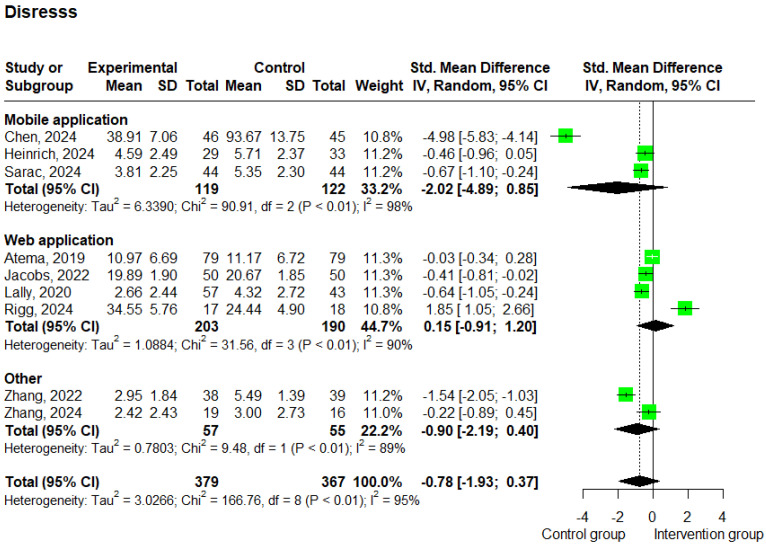
Forest plots—effects of e-health interventions on distress, presented by method of intervention (web application: *p* = 0.783; mobile application: *p* = 0.168; other: *p* = 0.173) [20,24,30,36,37,38,43,44,47]. SD, standard deviation; SMD, standardized mean difference. The “Other” category includes two studies that used virtual reality-based interventions (*p* = 0.1734).

A total of nine studies [20,24,30,36,37,38,43,44,47] reported data on distress. These studies included 746 patients and showed substantial heterogeneity (I^2^ = 95%). Compared to the control group, the analysis showed that the e-health intervention did not have a statistically significant impact on the intervention group (SMD: −0.78; 95% CI −1.93 to 0.37; *p* = 0.184; and I^2^ = 95%) (Figure 7).

The subgroup analysis based on the delivery method showed that studies using web-based interventions (*n* = 3) [38,41,43,44] yielded an SMD of 0.15 (95% CI: −0.91 to 1.20; *p* = 0.783; and I^2^ = 90%), while studies using mobile-based interventions (*n* = 3) [20,24,30] reported an SMD of −2.02 (95% CI: −4.89 to 0.85; *p* = 0.168; and I^2^ = 98%) (Figure 7). Furthermore, the subgroup analysis based on the intervention duration revealed that studies with a duration of less than 12 weeks (*n* = 3) [20,30,44] showed an SMD of −1.26 (95% CI: −5.16 to 2.63; *p* = 0.52; and I^2^ = 99%), while studies lasting 12 weeks or more (*n* = 6) [24,36,37,38,43,47] showed a statistically significant SMD of −0.54 (95% CI: −0.96 to −0.12; *p* = 0.011; and I^2^ = 81%) (Supplementary Figure S4). Additionally, it is important to note that low scores on the distress scales correspond to a lower level of distress.

Moreover, visual inspection of the funnel plots and Egger’s test analyses [19] indicated no significant publication bias. This assessment was performed only for anxiety, depression, and quality of life, as the number of studies included in each of these fields was 10 or more (Supplementary Figures S5–S7). A *p*-value < 0.1 was considered indicative of small study effects.

## 4. Discussion

Global adoption of new technologies and their rapid growth in popularity have had an important impact on patient care management, bringing exciting potential as well as significant challenges. Patients who have severe or chronic disorders frequently receive digital support at home in order to prevent, manage, and ameliorate disease symptoms and adverse treatment effects. Additionally, real-time remote interactions reduce the effort of attending hospital visits and reduce treatment delays and travel costs. Access to cancer treatment is improved by using e-health approaches, particularly for patients who live in remote areas, have difficulties traveling, or prefer to communicate from home [48]. Furthermore, it is important to mention that COVID-19 was a key contributor to the rapid adoption of e-health technologies, permanently integrating online medical services into the healthcare system and changing the way care is provided [49].

Alongside e-health interventions, artificial intelligence (AI) is rapidly transforming cancer care, offering an opportunity for significant improvements in both patient survival and quality of life [50]. Future AI-enhanced tools could provide clinicians with quick, cost-effective, and globally accessible solutions, helping match patients with the most appropriate treatments while simultaneously reducing potential negative impacts. AI methods will be able to integrate multiple data sources, such as patient-generated health data (PGHD) and electronic health records (EHRs), for personalized treatment recommendations and prediction of outcomes [51]. This precision-based approach could strengthen clinical decision-making, reduce time for treatment, and deliver personalized care tailored to each patient’s needs [52]. In addition, large language models (LLMs) have shown potential for classifying and predicting complex interactions, such as chemotherapy-induced toxicity, which could enhance patient monitoring and reduce treatment complications [53]. As AI technology evolves and becomes increasingly integrated into medicine, it holds the potential to further enhance cancer management, offering even more impressive outcomes for patient survival and well-being [54]. AI has significantly enhanced diagnostic capacities through data analysis and machine learning algorithms, increasing the accuracy of disease diagnosis and decreasing diagnostic errors [55].

According to our analysis, the experimental group’s anxiety symptoms were lower than those of the control group. The moderate effect, demonstrated by the SMD value of −0.80, indicates the efficacy of experimental interventions. Although the confidence range is quite broad (ranging from −1.33 to −0.27), it was still below zero, indicating that the experimental group derived significant benefits. The statistical significance of the result was confirmed by the low value of *p* (<0.01). This result should be interpreted with caution, as there was significant heterogeneity between studies (indicated by the high value of I^2^ = 94%), which raises the possibility that variables such as study design, demographics, or the variety of interventions used may have played a role in the credibility of the results observed. Moreover, the subgroup analysis based on the method of intervention delivery revealed that only mobile applications managed to reach statistical significance. Mobile applications have been extensively used in the literature because they offer ‘accessibility, convenience, and adaptability’, ‘patient-centeredness’, ‘data insights’, and ‘efficiency and effectiveness’ [56].

As with anxiety, the experimental group’s SMD score for depression symptoms was −0.74, indicating a substantial decrease in this symptom through the use of e-health support. The experimental group performed better because the confidence interval was negative, and it spanned from −1.40 to −0.09. The statistical significance of the outcome was confirmed by the *p*-value < 0.01. Again, the heterogeneity between the studies was high (I^2^ = 95%), which must be considered when interpreting the results, as various variables likely affected the findings. The subgroup analysis based on the method of intervention delivery confirmed that only web applications reached statistical significance in the context of depression. Furthermore, the literature confirms that web applications can reduce the burden of chronic mental illness and improve patient outcomes [57].

With an SMD value of 0.65, the experimental intervention was linked to a small but not negligible and statistically significant (*p* value < 0.01) improvement in the overall QoL. The positive impact of the intervention is indicated by the confidence interval (0.27 to 1.04) being above zero. However, an I^2^ score of 90% suggests high heterogeneity between the studies, which raises questions regarding the uniformity and comparability of the results across the studies.

The intervention reduced distress (SMD = −0.78), but the effect was unclear due to the wide confidence interval, which included zero (−1.93 to 0.37). Although the pattern generally favored the experimental group, it did not reach statistical significance (*p* = 0.184). Extreme heterogeneity was indicated by the very high I^2^ value of 95%, which suggests that variables like demographics, intervention techniques, or measuring instruments may have had a considerable impact on the outcomes. Also, it has to be considered that distress in cancer care is a multifactorial dimension, and the measures to quantify it may focus on different subdimensions [58,59].

The subgroup analyses showed that mobile-based interventions were more effective than web-based ones for anxiety and QoL, while only web-based interventions showed a significant effect on depression. Additionally, the subgroup analyses revealed that both short-term (less than 12 weeks) and long-term (12 weeks or more) interventions significantly improved quality of life and reduced anxiety in patients, with the best results observed in interventions shorter than 12 weeks, highlighting the importance of the intervention duration. Notably, the duration of the intervention also played an important role in distress, as patients receiving e-health interventions reported significantly lower distress than the control group after a longer duration (12 weeks or more) of intervention.

Furthermore, it is important to note that most subgroups had high levels of heterogeneity, indicating differences in the populations and study designs.

Notably, four of the five studies [27,35,36,44], whose findings indicated no beneficial effect on specific psychological factors, involved patients with metastatic cancer, a population that faces serious psychological difficulties as a result of the disease’s advanced stage. These studies’ lack of effectiveness may have been due to the group’s increased psychological stress, which makes improvement challenging through interventions that only address some of their psychological demands. The fifth study [23] used the Breast Cancer Patient Support System app for side-effect management by tracking symptoms during chemotherapy. While it offered some assistance, it was not enough to improve patients’ psychological well-being, which requires more comprehensive and intensive interventions.

To the best of our knowledge, this study marks a milestone as one of the pioneering efforts to systematically examine the impact of e-health interventions on the mental health and QoL of patients with BC. The findings of this systematic review highlight the importance of e-health interventions in improving the mental health and QoL of BC patients. The analysis of 27 RCTs, with a total sample size of 2898 patients, shows that e-health interventions can significantly reduce anxiety and depression while helping to improve QoL. Furthermore, these results align with earlier systematic reviews that suggest e-health interventions can enhance the mental health and QoL of BC patients [60,61]. Conversely, it is noteworthy that the e-health interventions failed to reduce the distress that BC patients were experiencing. However, the high heterogeneity (I^2^ > 85%) of the findings underscores the need for further standardization in e-health interventions, even with the favorable impact. Comparing interventions that differ in duration, frequency, and structure, as well as in how they monitor results, is challenging and makes determining their overall efficacy and interpretation extremely difficult. Furthermore, it is difficult to define the specific variables that lead to the most significant patient outcomes, since there are no generally recognized criteria. An integrated approach to intervention planning and implementation is required to increase the reliability of future studies.

### Limitations

This study has certain limitations: (i) The search strategy was limited to studies published in English. (ii) There was diversity in the frequency, content, and delivery formats of e-health interventions. (iii) In some studies, the number of patients included was small, which raises concerns about the reliability and generalizability of the results. (iv) The effectiveness of each intervention may have been affected by a possible low level of patient adherence. It is essential to acknowledge these limitations and putative influencing factors as reasons for the surprisingly non-positive results for the many studied domains. Therefore, to define the optimal type and regimen of e-health intervention, high-quality RCTs are needed.

## 5. Conclusions

E-health support has been shown to significantly improve QoL and reduce anxiety and depression in BC patients. These results imply that e-health interventions have been at least to some extent successful and beneficial. Considering the decades-long dominance of traditional treatments and supportive care management, these e-health intervention outcomes are more than encouraging for the future of medical care. Additionally, the reasons for the moderate effectiveness of some e-health interventions need to be further analyzed, including potential biases, implementation issues, and methodological weaknesses that may impact the results.

## Figures and Tables

**Table 1:** Inclusion criteria for studies.

Parameter	Inclusion Criteria
Population	Adult men or women (aged > 18 years old) diagnosed with BC
Intervention	Patient-directed e-health intervention
Comparator	Studies in which patients received standard care or control intervention
Outcomes	QoL, anxiety, depression, distress
Study Design	Randomized controlled trials

**Table 2:** Studies on e-health interventions in cancer patients.

Authors	PMID/DOI	Number of Patients	Stage/Status	Therapy	Experimental Intervention	Comparison	Duration of Intervention (Weeks)	Study Outcomes (Compared to Control Group)
Akkol-Solakoglu, et al. [46]	36635249	Total = 72 (I = 49, C = 23), Mean age: 47.8	0, I, II, III, IV	Chemotherapy, Radiotherapy, Hormonal therapy, Surgery	Web-based cognitive behavioral therapy	Usual care	8	No significant effect on anxiety, depression, fear of recurrence, and QoL.
Atema et al. [38]	30763176	Total = 169 (I = 85, C = 85), Mean age: 47.4	I, II, III, IV	Surgery, Chemotherapy, Radiation therapy, Immunotherapy, Endocrine therapy, Oophorectomy	Internet-based cognitive behavioral therapy	Waiting list	24	Improvements in hot flushes, sleep quality, and menopausal symptoms.
Chen et al. [20]	38889503	Total = 94 (I = 47, C = 47), Mean age: 49.3	I, II	Chemotherapy	Phone-based support program	Usual care	7	Higher self-care efficacy, better QoL, less symptom distress, reduced anxiety and depression.
Dong et al. [21]	31242926	Total = 60 (I = 30, C = 30), Mean age: 49.7	I, II, III	Chemotherapy	Internet and social media software (CEIBISMS)	Traditional rehab care	12	Improvements in vitality, mental health, and health transition.
Ghanbari et al. [22]	34003138	Total = 82 (I = 41, C = 41), Mean age: 46.4	Nonmetastatic	Not reported	mHealth psychoeducational intervention	Waiting list	5	Lower anxiety and higher self-esteem.
Graham et al. [39]	38752788	Total = 79 (I = 40, C = 39), Mean age: 59.4	I, II, III	Surgery, Chemotherapy, Radiation therapy, Hormone therapy	Remotely delivered one-to-one therapy	Usual care	24	Improvements in medication adherence, QoL, distress, and flexibility.
Handa et al. [23]	32201165	Total = 102 (I = 52, C = 50), Mean age: 49.9	ER+, ER-, PR+, PR-, HER2+, HER2-	Chemotherapy	Smartphone app during chemotherapy	Usual care	12	No significant anxiety/depression change; possible enhanced care via info sharing.
Heinrich et al. [24]	39439014	Total = 70 (I = 32, C = 38), Mean age: 57.6	Primary breast cancer	Surgery, Chemotherapy, Radiation therapy	mHealth cognitive behavioral therapy	Usual care	12	Improved anxiety, HRQoL, and illness perception.
Holtdirk et al. [40]	33961667	Total = 363 (I = 181, C = 182), Mean age: 49.9	Not reported	Surgery, Chemotherapy, Radiation treatment	Website with CBT	Usual care	12	Improved QoL and diet; no change in exercise.
Jacobs et al. [41]	35924869	Total = 100 (I = 50, C = 50), Mean age: 56.1	0, I, II, III	Surgery, Chemotherapy, Radiation therapy, Endocrine therapy	Telehealth for symptom management	Medication monitoring	12	Less distress, better self-management, coping, mood, and QoL.
Kim et al. [35]	30578205	Total = 76 (I = 36, C = 40), Mean age: 51.0	IV	Chemotherapy (taxanes, anthracyclines, capecitabine, platinum compounds)	mHealth game to reduce chemotherapy side effects	Conventional education group	3	Better drug adherence, fewer chemotherapy adverse effects, better QoL, no significant difference in depression or anxiety.

**Table 3:** Studies on e-health interventions in cancer patients.

Authors	PMID/DOI	Number of Patients	Stage/Status	Therapy	Experimental Intervention	Comparison	Duration of Intervention (Weeks)	Study Outcomes (Compared to Control Group)
Korkmaz et al. [42]	31119709	Total = 48 (I = 24, C = 24), Mean age: 47.7	II, III	Surgery	Web-based education program on anxiety and QoL	Routine education	4	Lower levels of anxiety and improvements in QoL.
Lally et al. [43]	31414245	Total = 100 (I = 57, C = 43), Mean age: 54.2	0, I, II	Surgery, Chemotherapy, Radiation therapy	Tailored self-management psychoeducational program	Usual care	12	No significant outcomes.
Li et al. [25]	39363984	Total = 44 (I = 23, C = 21), Mean age: 47.9	I, II, III	Chemotherapy	Wearable device-based aerobic exercise for physical and mental health	Waiting list	12	Improvements in physical fitness, mental health, sleep quality, QoL, and fewer adverse effects.
Okuyama et al. [26]	38796818	Total = 125 (I = 61, C = 64), Mean age: 63.5	I, II, III	Chemotherapy, Radiotherapy, endocrine therapy, Combination therapy	Electronic patient-reported outcome app	Usual care	12	No improvements in BC patients’ QoL.
Philips et al. [27]	39014267	Total = 49 (I = 25, C = 24), Mean age: 54.8	IV	Chemotherapy, Radiation therapy, Immunotherapy, Targeted therapy, Hormone therapy	Physical activity promotion via mHealth intervention	Healthy lifestyle control	12	Improvements in activity, QoL, some PROs, social cognitive theory constructs, and functional performance.
Peng et al. [28]	36347151	Total = 60 (I = 30, C = 30), Mean age: 41.8	I, II, III, IV	mastectomy, conservative therapy, mastectomy + breast construction	Online mindfulness-based intervention on fear of cancer recurrence and quality of life	Usual care	6	Lower level of fear of cancer recurrence (FCR) and an improvement in quality of life
Rigg et al. [44]	39438337	Total = 35 (I = 17, C = 18), Mean age: 57.4	IV	Surgery, Chemotherapy, Radiotherapy, Hormonal therapy, Other treatment	Web-based self-guided psychosocial program	Usual care	6	Small improvements in fear of progression and global QoL, alongside some deteriorations in distress and mental QoL.
Rosen et al. [29]	10.1002/pon.4764	Total = 112 (I = 57, C = 55), Mean age: 52.2	Not reported	Not reported	mHealth mindfulness training	Waiting list	8	Improvements in QoL.
Sarac et al. [30]	39257013	Total = 82 (I = 42, C = 40), Mean age: 49.0	Not reported	Adjuvant, Neoadjuvant, Surgery (BCS + SLNB, Mastectomy + SLNB, MRM)	Informative mobile app use on anxiety, distress, and QoL	Usual care	4	Lower anxiety and distress levels, but no difference in overall QoL.
Singleton et al. [31]	35460441	Total = 156 (I = 78, C = 78), Mean age: 55.1	Not reported	Surgery, Radiotherapy, Chemotherapy, Endocrine therapy, Targeted therapy	Supporting women’s health outcomes through text messages.	Usual care	24	No significant differences between groups for self-efficacy, adjusted mean difference, QoL, mental health, physical activity, or BMI.

**Table 4:** Studies on e-health interventions in cancer patients.

Authors	PMID/DOI	Number of Patients	Stage/Status	Therapy	Experimental Intervention	Comparison	Duration of Intervention (Weeks)	Study Outcomes (Compared to Control Group)
White et al. [45]	30137657	Total = 337 (I = 202, C = 177), Mean age: 43.7	I, II	Surgery, Chemotherapy, Radiotherapy, Targeted therapy, Hormonal therapy	Information-based breast cancer-specific website	Usual care	24	Mean level of QoL scores did not differ between groups.
Zhang et al. [36]	38418478	Total = 36 (I = 19, C = 17), Mean age: 47.2	IV	Not reported	Virtual reality intervention for managing cancer and living meaningfully.	Waiting list	12	CALM therapy led to reductions in depression, distress, and attachment avoidance, as well as improvements in quality of life.
Zhang et al. [37]	35712124	Total = 90 (I = 45, C = 45), Mean age: 51.6	I, II, III, IV	Surgery, Chemotherapy	Virtual reality intervention for psychological distress and symptom management.	Usual care	12	VR-CALM improves well-being in survivors.
Zhou et al. [32]	32272281	Total = 111 (I = 56, C = 55), Mean age: 49.9	I, II, III	Surgery, Chemotherapy, Radiotherapy, Endocrine therapy	WeChat-based nursing program for postoperative BC rehabilitation	Usual care	24	Significant improvement in HRQoL.
Zhou et al. [34]	31342310	Total = 132 (I = 66, C = 66), Mean age: 44.5	I, II, III	Surgery, Chemotherapy, Radiotherapy, Endocrine therapy	Mobile-based training on resilience, depression, and anxiety management	Usual care	12	Improvements were observed in psychological resilience, anxiety, and depression scores.
Zhu et al. [33]	29712622	Total = 114 (I = 57, C = 57), Mean age: 47.2	I, II, III, IV	Surgery, Chemotherapy	Mobile breast cancer e-support program	Usual care	12	E-support + care improved self-efficacy, symptom interference, and QoL but not social support, symptom severity, anxiety, or depression.

## Data Availability

The data presented in this study are available in this article and Supplementary Materials.

## Supplementary Materials

The following supporting information can be downloaded at https://www.mdpi.com/article/10.3390/cancers17111780/s1, Figure S1: Forest plots showing the effects of e-Health interventions on anxiety, presented by intervention duration (less than 12 weeks: *p* = 0.011; 12 weeks or more: *p* = 0.033); Figure S2: Forest plots showing the effects of e-Health interventions on depression, presented by intervention duration (less than 12 weeks: *p* = 0.278; 12 weeks or more: *p* = 0.065); Figure S3: Forest plots showing the effects of e-Health interventions on quality of life (QoL), presented by intervention duration (less than 12 weeks: *p* = 0.022; 12 weeks or more: *p* = 0.012); Figure S4: Forest plots showing the effects of e-Health interventions on distress, presented by intervention duration (less than 12 weeks: *p* = 0.52; 12 weeks or more: *p* = 0.011); Figure S5: Funnel plot of publication bias on anxiety. The result of Egger’s test (*p* = 0.003) indicates the presence of small study effects, as suggested by the asymmetry in the plot; Figure S6: funnel plot of publication bias on depression. The result of Egger’s test (*p* = 0.037) indicates the presence of small study effects, as suggested by the asymmetry in the plot; Figure S7: Funnel plot of publication bias on QoL. The result of Egger’s test (*p* = 0.032) indicates the presence of small study effects, as suggested by the asymmetry in the plot.

## Abbreviations

The following abbreviations are used in this manuscript:

**Abbreviation**	**Definition**
App	Application
BC	Breast cancer
E-health	Electronic health
HRQoL	Health-related quality of life
IARC	International Agency for Research on Cancer
PICOS	Population, Intervention, Comparator, Outcomes, Study Design
PRISMA	Preferred Reporting Items for Systematic Reviews and Meta-Analysis
QoL	Quality of life
RCT	Randomized controlled trial
AI	Artificial intelligence
PGHD	Patient-generated health data
EHRs	Electronic health records
