# Supplementary material for: Impact of e-Health Interventions on Mental Health and Quality of Life in Breast Cancer Patients: A Systematic Review and Meta-Analysis of Randomized Controlled Trials
Source: Cancers (Basel). 2025 May 26;17(11):1780. doi: 10.3390/cancers17111780 (PMC12153910; doi:10.3390/cancers17111780)
Supplement: Supplementary file 1 [file cancers-17-01780-s001.zip › cancers-3592581-supplementary.pdf]

Anxiety

Study or Subgroup	Experimental Mean	SD	Total	Control Mean	SD	Total	Weight	Std. Mean Difference IV, Random, 95% CI
**Duration of intervention: less than 12 weeks**								
Akkol-Solakoglu, 2023	8.53	4.60	38	10.79	4.01	19	5.2%	-0.50 [-1.06; 0.05]
Chen, 2024	4.89	1.64	46	10.00	2.52	45	5.2%	-2.39 [-2.93; -1.85]
Ghanbari, 2021	90.66	13.84	38	106.92	15.94	39	5.3%	-1.08 [-1.56; -0.60]
Kim, 2018	40.60	3.60	34	42.00	3.80	38	5.3%	-0.37 [-0.84; 0.09]
Sarac, 2024	4.77	4.04	44	6.55	4.04	44	5.3%	-0.44 [-0.86; -0.01]
**Total (95% CI)**			**200**			**185**	**26.4%**	**-0.95 [-1.69; -0.21]**
Heterogeneity: Tau^2 = 0.6407; Chi^2 = 40.8, df = 4 (P < 0.01); I^2 = 90%								
**Duration of intervention: 12 weeks or more**								
Atema, 2019	6.53	3.92	79	6.53	3.94	79	5.4%	0.00 [-0.31; 0.31]
Graham, 2024	3.10	3.50	35	4.70	3.60	31	5.3%	-0.45 [-0.94; 0.04]
Holtdirk,2021	6.83	4.02	141	7.20	4.39	165	5.5%	-0.09 [-0.31; 0.14]
Jacobs, 2022	6.54	0.93	50	6.59	0.92	50	5.4%	-0.05 [-0.45; 0.34]
White, 2018	6.20	3.70	157	6.50	4.10	142	5.5%	-0.08 [-0.30; 0.15]
Zhang, 2022	44.16	11.08	38	55.21	3.81	39	5.3%	-1.33 [-1.82; -0.83]
Zhang, 2024	28.42	12.03	19	27.13	11.41	16	5.1%	0.11 [-0.56; 0.77]
Handa, 2020	1.66	2.58	47	0.46	3.12	48	5.4%	0.42 [0.01; 0.82]
Heinrich, 2024	8.91	2.21	29	10.09	2.15	33	5.3%	-0.53 [-1.04; -0.03]
Li, 2024	31.76	1.27	21	37.00	1.49	19	4.5%	-3.73 [-4.78; -2.67]
Phillips, 2024	48.80	1.90	25	51.00	1.90	24	5.2%	-1.14 [-1.75; -0.53]
Singleton, 2023	19.30	15.50	78	20.10	14.60	78	5.4%	-0.05 [-0.37; 0.26]
Zhou, 2019	22.21	2.87	60	34.12	3.05	58	5.1%	-4.00 [-4.63; -3.37]
Zhu, 2018	9.93	2.72	57	10.28	2.46	57	5.4%	-0.13 [-0.50; 0.23]
**Total (95% CI)**			**836**			**839**	**73.6%**	**-0.75 [-1.45; -0.06]**
Heterogeneity: Tau^2 = 1.6833; Chi^2 = 225.45, df = 13 (P < 0.01); I^2 = 94%								
**Total (95% CI)**			**1036**			**1024**	**100.0%**	**-0.80 [-1.33; -0.27]**
Heterogeneity: Tau^2 = 1.3424; Chi^2 = 290.22, df = 18 (P < 0.01); I^2 = 94%								
Test for subgroup differences: Chi^2 = 0.14, df = 1 (P = 0.70)								

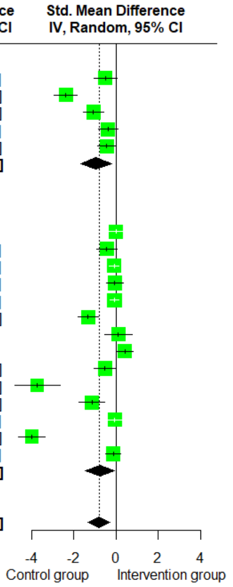

Figure S1: *Forest plots showing the effects of e-Health interventions on anxiety, presented by intervention duration (less than 12 weeks: p = 0.011; 12 weeks or more: p = 0.033). SD, standard deviation; SMD, standardized mean difference.*

Depression

Study or Subgroup	Experimental Mean	SD	Total	Control Mean	SD	Total	Weight	Std. Mean Difference IV, Random, 95% CI
**Duration of intervention: less than 12 weeks**								
Akkol-Solakoglu, 2023	4.26	3.48	38	5.63	3.24	19	6.2%	-0.40 [-0.95; 0.16]
Chen, 2024	3.65	1.57	46	10.36	2.30	45	6.1%	-3.39 [-4.03; -2.74]
Kim, 2018	15.70	3.70	34	14.90	5.20	38	6.3%	0.17 [-0.29; 0.64]
**Total (95% CI)**			**118**			**102**	**18.6%**	**-1.19 [-3.35; 0.96]**
Heterogeneity: Tau^2 = 3.5504; Chi^2 = 79.91, df = 2 (P < 0.01); I^2 = 97%								
**Duration of intervention: 12 weeks or more**								
Atema, 2019	4.44	3.47	79	4.64	3.48	79	6.4%	-0.06 [-0.37; 0.25]
Graham, 2024	3.90	3.20	34	5.00	3.10	34	6.3%	-0.35 [-0.82; 0.13]
Holtdirk,2021	7.06	4.23	141	8.38	4.77	165	6.5%	-0.29 [-0.52; -0.06]
Jacobs, 2022	3.87	0.70	50	4.26	0.70	50	6.3%	-0.55 [-0.95; -0.15]
White, 2018	3.40	3.50	157	3.50	3.60	142	6.5%	-0.03 [-0.26; 0.20]
Zhang, 2022	46.63	9.82	38	50.21	3.81	39	6.3%	-0.48 [-0.93; -0.02]
Zhang, 2024	8.95	6.45	19	9.63	7.14	16	6.1%	-0.10 [-0.76; 0.57]
Handa, 2020	0.09	2.74	47	-0.42	2.79	48	6.3%	0.18 [-0.22; 0.59]
Heinrich, 2024	10.19	2.26	29	11.00	2.32	33	6.3%	-0.35 [-0.85; 0.15]
Li, 2024	34.57	1.67	21	40.74	2.29	19	5.7%	-3.04 [-3.98; -2.11]
Phillips, 2024	46.40	1.50	25	46.00	1.50	24	6.2%	0.26 [-0.30; 0.83]
Zhou, 2019	22.87	2.59	60	34.41	3.20	58	6.1%	-3.95 [-4.57; -3.32]
Zhu, 2018	12.75	1.57	57	12.58	2.15	57	6.4%	0.09 [-0.28; 0.46]
**Total (95% CI)**			**757**			**764**	**81.4%**	**-0.64 [-1.31; 0.04]**
Heterogeneity: Tau^2 = 1.4827; Chi^2 = 187.27, df = 12 (P < 0.01); I^2 = 94%								
**Total (95% CI)**			**875**			**866**	**100.0%**	**-0.74 [-1.40; -0.09]**
Heterogeneity: Tau^2 = 1.7084; Chi^2 = 277.44, df = 15 (P < 0.01); I^2 = 95%								
Test for subgroup differences: Chi^2 = 0.23, df = 1 (P = 0.63)								

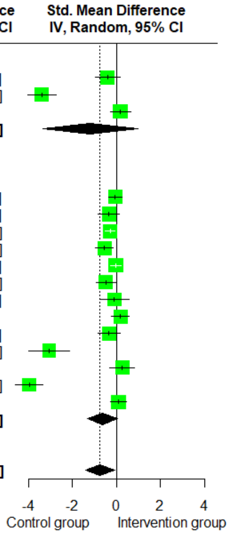

Figure S2: *Forest plots showing the effects of e-Health interventions on depression, presented by intervention duration (less than 12 weeks: p = 0.278; 12 weeks or more: p = 0.065). SD, standard deviation; SMD, standardized mean difference.*

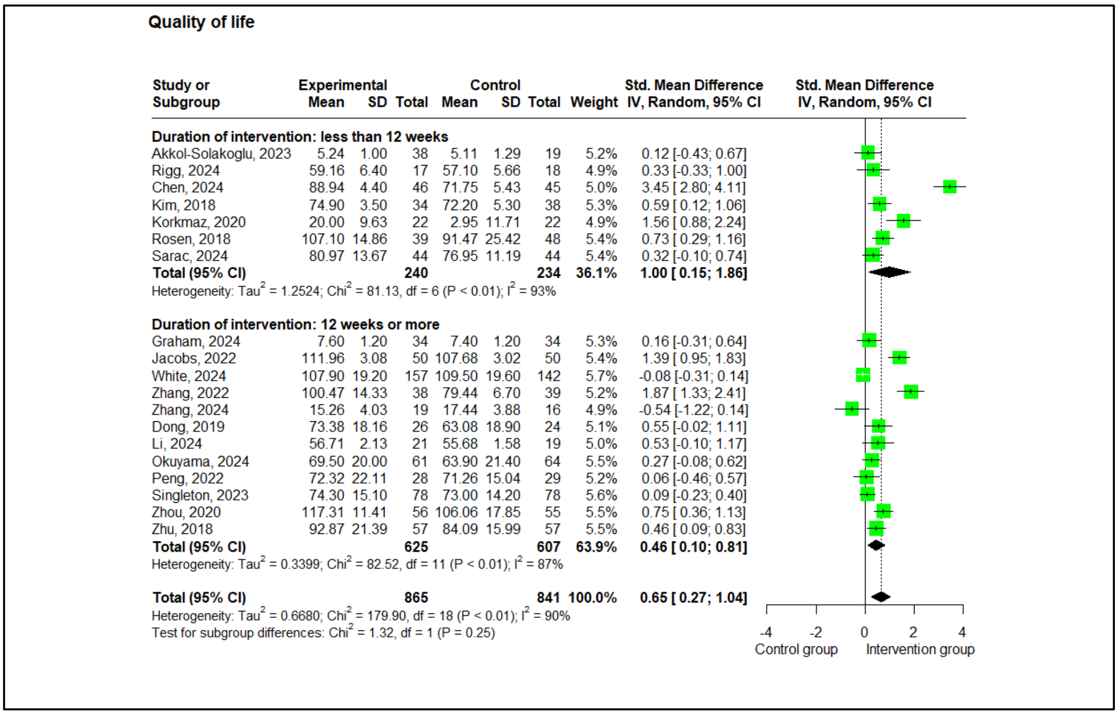

Figure S3*: Forest plots showing the effects of e-Health interventions on quality of life (QoL), presented by intervention duration (less than 12 weeks: p = 0.022; 12 weeks or more: p = 0.012). SD, standard deviation; SMD, standardized mean difference.*

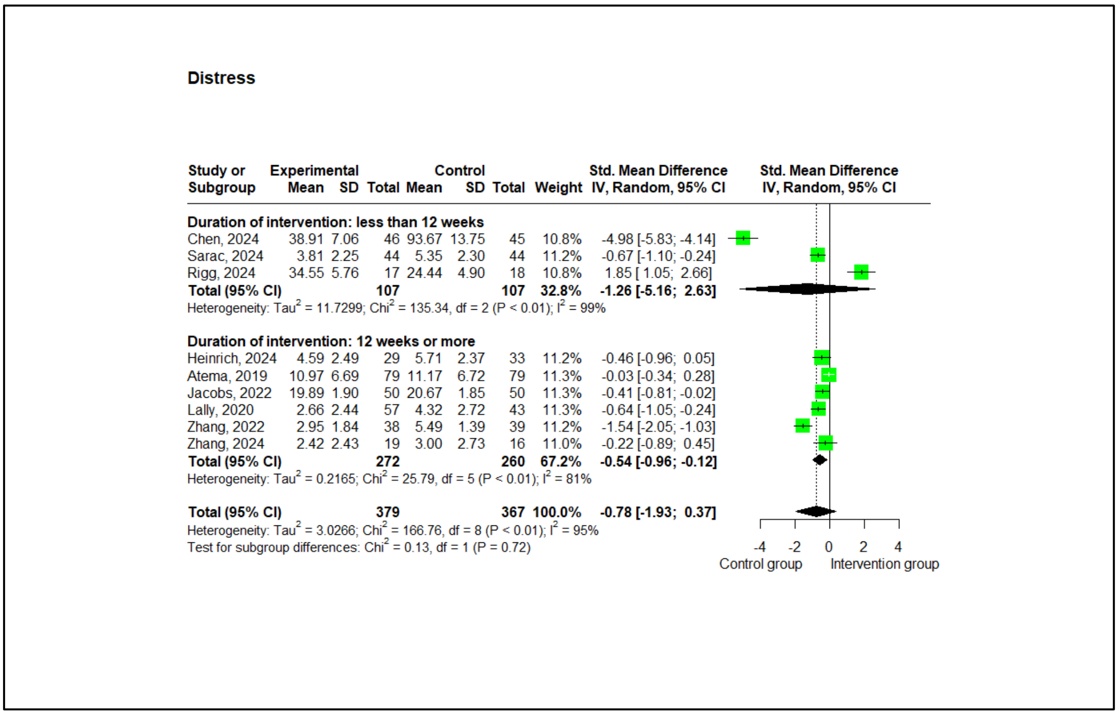

Figure S4: *Forest plots showing the effects of e-Health interventions on distress, presented by intervention duration (less than 12 weeks: p = 0.52; 12 weeks or more: p = 0.011). SD, standard deviation; SMD, standardized mean difference.*

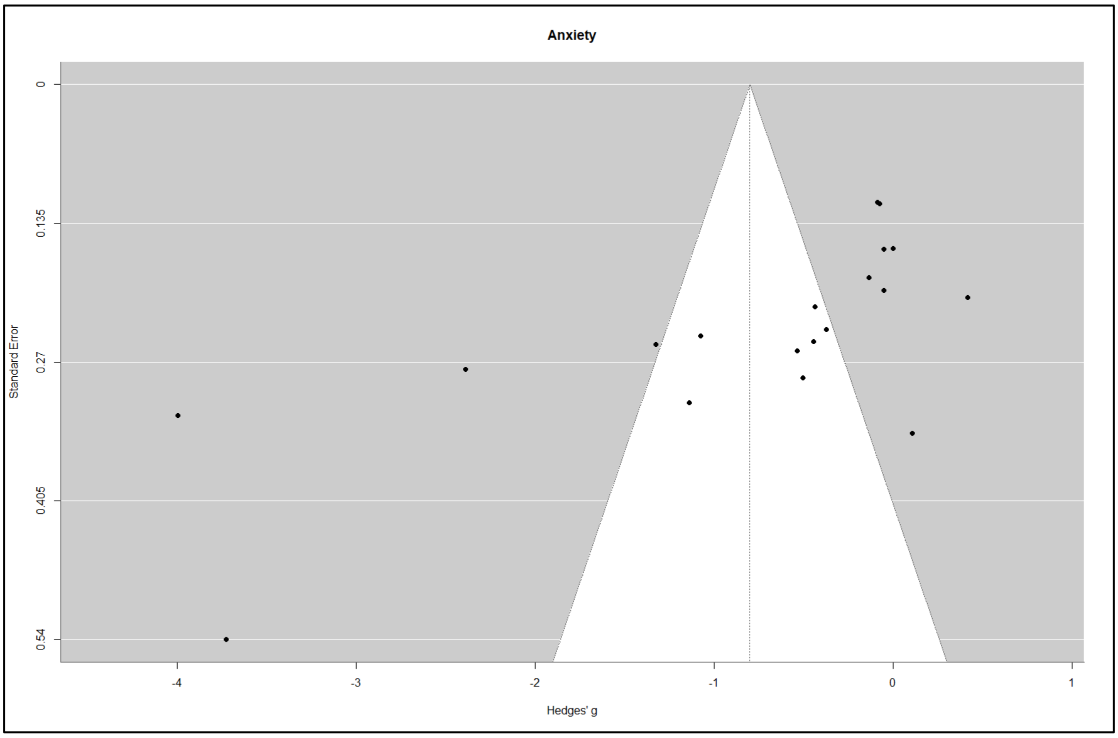

Figure S5*: Funnel plot of publication bias on anxiety. The result of Egger's test (p = 0.003) indicates the presence of small study effects, as suggested by the asymmetry in the plot.*

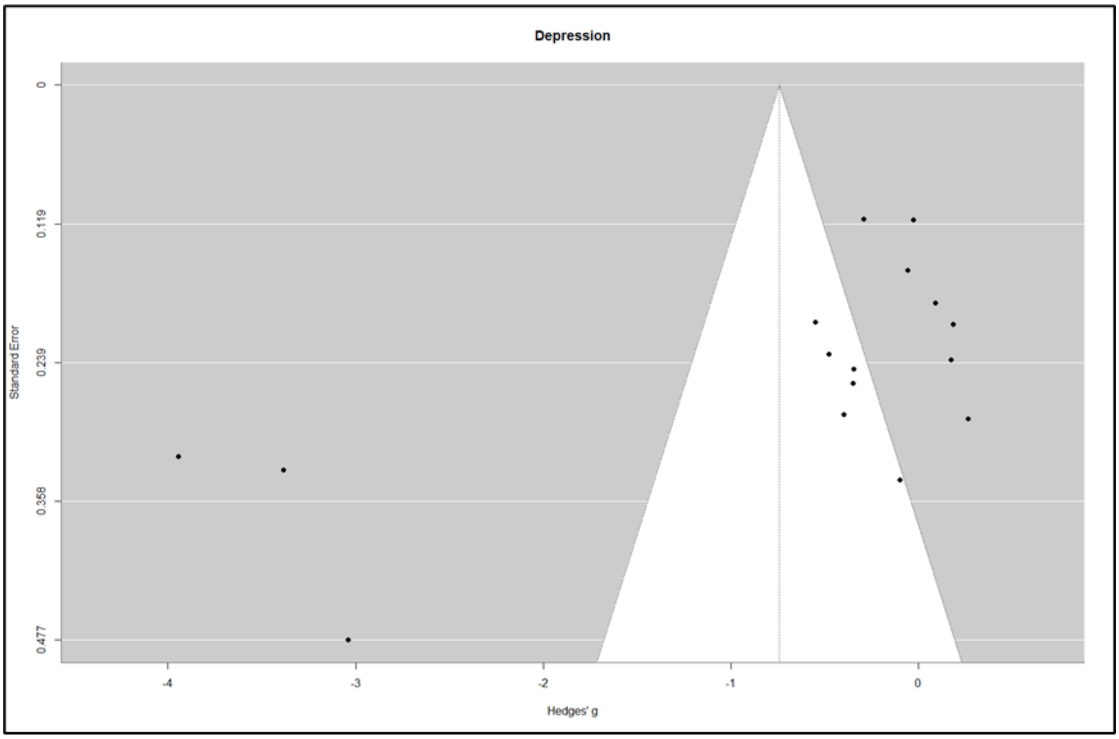

Figure S6: *funnel plot of publication bias on depression. The result of Egger's test (p = 0.037) indicates the presence of small study effects, as suggested by the asymmetry in the plot.*

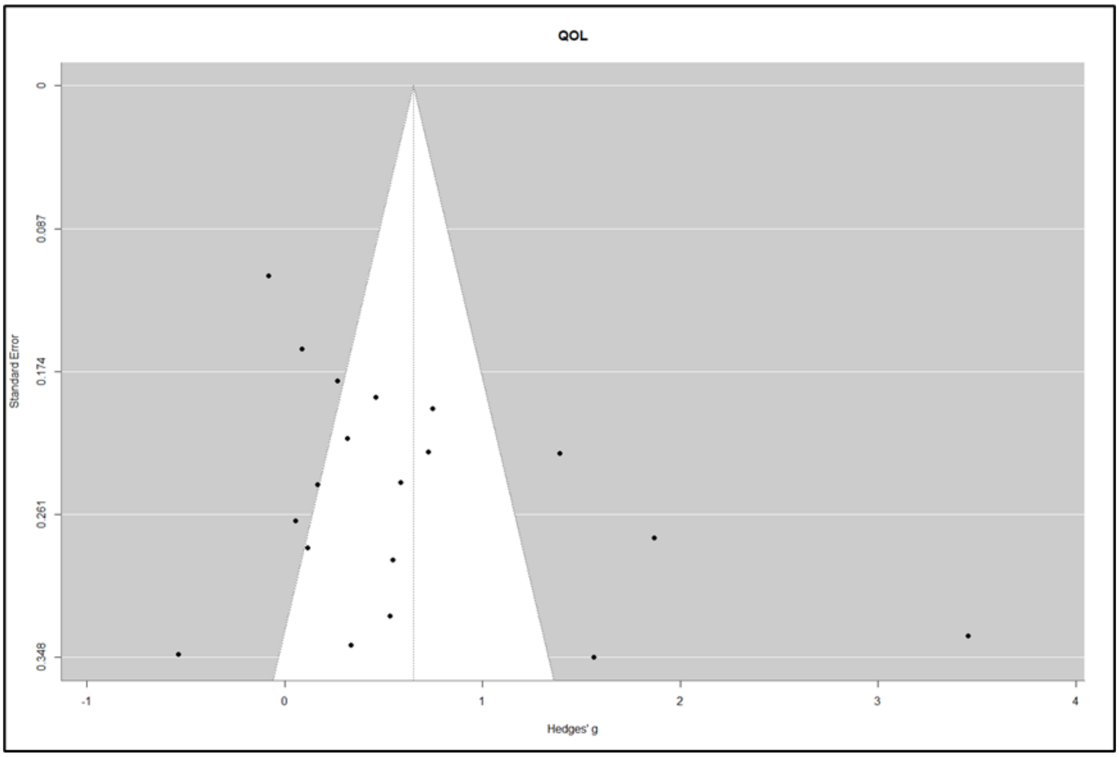

Figure S7: *Funnel plot of publication bias on QoL. The result of Egger's test (p = 0.032) indicates the presence of small study effects, as suggested by the asymmetry in the plot.*
